# Assessing LV Contractility Identifies Populations With Preserved Ejection Fraction at Risk of Adverse Heart Failure Outcomes

**DOI:** 10.1161/CIRCIMAGING.125.018370

**Published:** 2025-07-16

**Authors:** Sam Straw, Oliver I. Brown, Charlotte A. Cole, Judith E. Lowry, Marcella Conning-Rowland, Stephe Kamalathasan, Sushma Datla, Maria F. Paton, Ruth Burgess, Michael Drozd, Thomas A. Slater, Samuel D. Relton, Eylem Levelt, Klaus K. Witte, Mark T. Kearney, Richard M. Cubbon, John Gierula

**Affiliations:** 1 Leeds Institute of Cardiovascular and Metabolic Medicine (S.S., O.I.B., M.C.-R., M.F.P., M.D., T.A.S., E.L., K.K.W., M.T.K., R.M.C., J.G.), University of Leeds, United Kingdom.; 2 Leeds Institute for Data Analytics (S.D.R.), University of Leeds, United Kingdom.; 3 Leeds Teaching Hospitals NHS Trust, United Kingdom (C.A.C., J.E.L., S.K., R.B.).; 4 York and Scarborough Teaching Hospitals, United Kingdom (S.D.).; 5 Baker Heart and Diabetes Institute, Melbourne, Australia (E.L.).

**Keywords:** blood pressure, heart failure, humans, prognosis, stroke volume

## Abstract

**BACKGROUND:**

Left ventricular ejection fraction (LVEF) is an essential tool for heart failure (HF) assessment but is limited by load dependence. Additional tools are needed to risk-stratify normal LVEF populations. We aimed to assess the prognostic value of systolic blood pressure-indexed left ventricular end-systolic volume ratio, or cardiac contractility index (CCI).

**METHODS:**

In a prospective observational cohort study of people newly diagnosed with HF, we defined characteristics and outcomes associated with LVEF and CCI, including after stratification into HF with reduced ejection fraction or HF with preserved ejection fraction. We used UK Biobank to assess whether CCI is associated with subclinical myocardial dysfunction and incident HF.

**RESULTS:**

In people with HF, mortality increased over tertiles of declining CCI (*P*<0.001). Within the HF with preserved ejection fraction group, below-median CCI was associated with distinct clinical characteristics and an all-cause mortality risk approximately twice that of those with above median CCI (observed event rate 17.3/100 patient-years versus 8.8/100 patient-years; *P*<0.001), similar to those with HF with reduced ejection fraction. Modeled as continuous variables, there was a curvilinear relationship between mortality across the detected range of CCI, while there was no clear association with mortality risk across a wide range of LVEF (20%–55%). In UK Biobank for participants without HF and normal LVEF, below-median CCI was associated with ≈33% increased risk of incident HF (adjusted hazard ratio, 1.33 [1.01–1.75]; *P*=0.043). Decreasing CCI was also associated with lower myocardial contractility defined using global radial and circumferential strain.

**CONCLUSIONS:**

CCI is a simple, noninvasive, relatively afterload-independent method to stratify HF risk in populations with normal LVEF. Its simplicity means CCI could be applied to existing clinical trial data sets or used be as an inclusion criterion in future randomized controlled trials.

**Clinical Perspective**

Current recommendations classify heart failure (HF) by left ventricular (LV) ejection fraction, largely based on the inclusion criteria of randomized controlled trials. There are well-known limitations of LV ejection fraction, including modest reproducibility, load dependence, and the fact that this measure represents the percentage change in LV volumes during the cardiac cycle, rather than contractility. LV ejection fraction also provides little prognostic information within those with HF with preserved ejection fraction. The ratio of systolic blood pressure to end-systolic volume index, or cardiac contractility index, provides additional information that helps reclassify individuals with HF with preserved ejection fraction. Below-median cardiac contractility index identifies a group with distinct clinical characteristics who are at increased risk of adverse clinical outcomes. Below-median cardiac contractility index also identifies healthy individuals without HF and apparently normal heart function who are at increased risk of incident HF. Assessing LV contractility may help identify patients with HF with preserved ejection fraction who could benefit from targeted therapies.

**See Editorial by Barasch and Khalique**

Heart failure (HF) is a syndrome characterized by breathlessness, fatigue, frequent hospitalization, and premature death. Current recommendations classify individuals by left ventricular ejection fraction (LVEF), the most commonly reported imaging biomarker of systolic function.^1^ Around half of people with HF have a preserved ejection fraction (HFpEF), and these individuals have similar symptoms and impairments in quality of life as those with HF with a reduced ejection fraction (HFrEF).^2^ LVEF is a complex summary measure of systolic function with known limitations, including modest reproducibility, load dependence, and represents the percentage change in left ventricular (LV) volume, rather than contractility.^3^

Although diastolic dysfunction is a key mechanism underpinning the pathophysiology of HFpEF, the presence of subtle or concomitant contractile dysfunction is an additional putative mechanism associated with adverse outcomes.^4^ Simply classifying individuals as having an LVEF that is normal may risk misclassifying individuals who have any degree of systolic dysfunction. While the ability to more easily identify subtle contractile dysfunction could help further refine the phenotypic classification of HFpEF, stratify risk, and identify those who have the potential to derive benefit from disease-modifying pharmacological therapies.

The ratio of LV end-systolic pressure to LV end-systolic volume provides a relatively afterload-independent assessment of LV contractility.^5^ An entirely noninvasive assessment of LV contractility is possible by using systolic blood pressure as a surrogate of LV end-systolic pressure.^6,7^ The ratio of systolic blood pressure to LV end-systolic volume index, or cardiac contractility index (CCI), is an accepted method of determining the force-frequency relationship in humans;^8–10^ however, its prognostic significance in unselected populations is unknown.

## Aims

First, we sought to evaluate the relationship between LV contractility and laboratory and imaging biomarkers of systolic function. Second, we aimed to evaluate whether LV contractility was associated with adverse clinical outcomes and whether this provided additional prognostic information beyond LVEF. Third, we explored whether assessing LV contractility could reclassify individuals with newly diagnosed HFpEF, and if these individuals had distinct characteristics and risk of adverse outcomes. Finally, we studied a population without HF and a normal LVEF to assess whether impaired LV contractility identified individuals at increased risk of incident HF.

## Methods

Detailed information regarding the study procedures, imaging acquisition and analysis, ascertainment of outcomes, and statistical analysis are described in full within the Supplementary Material.

### Data Availability Statement

Data sets generated and analyzed from the NICE-CHF (National Institute for Clinical Excellence Guidelines on Chronic Heart Failure) study are not publicly available due to the inclusion of potentially identifiable information but are available from the corresponding author upon reasonable request. The UK Biobank resource is open to all bona fide researchers. This analysis was conducted under application number 106965: Defining novel measures of LV contractility and their association with the risk of incident cardiovascular disease. Full details of its design and conduct are available online (https://www.ukbiobank.ac.uk).

### NICE-HF Cohort

#### Study Population

The prospective evaluation of the diagnostic efficacy of the 2010 United Kingdom NICE-CHF is an observational cohort study of people who were newly diagnosed with HF and classified according to currently applied definitions.^1^ This includes all individuals referred between May 2012 and May 2013 from a primary care catchment of over 750 000, with signs/symptoms of HF and elevated natriuretic peptides (NT-proBNP [N-terminal pro-B type natriuretic peptide] ≥125 pg/mL).^11^

#### Participant Classification and Outcomes

For simplicity, we regarded those with LVEF <50% as having HFrEF and those with LVEF ≥50% and relevant structural heart disease (either left atrial dilatation, LV hypertrophy) or diastolic dysfunction as having HFpEF. We also did sensitivity analyses in which we divided patients with abnormal LVEF into those with mildly reduced (40%–49%) and reduced (<40%) LVEF. Individuals without these echocardiographic features, including those in whom symptoms were attributable to significant valvular disease, were excluded. The primary outcome was all-cause mortality. Secondary outcomes were cardiovascular death and HF hospitalization. We also examined the association between CCI and laboratory and imaging biomarkers of systolic function, including LVEF.

#### Statistical Analysis

We compared the clinical characteristics and outcomes across tertiles of CCI and LVEF measured by transthoracic echocardiography. We also stratified individuals into 4 groups by whether they had HFrEF or HFpEF, and whether CCI was above or below the median value. Scatter plots were constructed for CCI, compared with LVEF, with correlation determined by Pearson correlation coefficients (*r*) and the coefficient of determination (*R*^2^). We plotted Kaplan-Meier curves to illustrate all-cause mortality rates, with significance testing by log-rank test. We used unadjusted and adjusted Poisson regression models to determine the association between CCI and LVEF with all-cause mortality, cardiovascular mortality, and HF hospitalization and presented these associations using restricted cubic splines. We also conducted a competing risks analysis using a Fine and Grey competing risks regression model for our secondary outcome, treating cardiovascular mortality as the outcome of interest, and noncardiovascular mortality as the competing event. Statistical analyses were done using Stata/MP (version 16.1, StataCorp LLC, College Station, TX) and R (version 4.1.1), with figures illustrated using PRISM (version 9, GraphPad Software, Inc, San Diego, CA). All tests were 2-sided and statistical significance was regarded as *P*<0.05.

#### Ethical Considerations

The United Kingdom Health Research Authority provided ethical approval through a Section 251 application reviewed by the Confidential Advisory Group (CAG8-03(PR1)/2013). Approval through a Section 251 application allows individual patient data to be used for health service improvement or medical research while waiving the requirement for individual patient consent. The project complied with the principles outlined in the Declaration of Helsinki.

### UK Biobank Cohort

#### Study Population

To conduct further analyses in people with a normal LVEF, we used data from UK Biobank, a prospective observational cohort study. This recruited 502 462 participants aged 37 to 73 years from 22 assessment centers across the United Kingdom between 2006 and 2010. At the time of analysis, data were available for ≈40 000 participants who subsequently underwent a standardized cardiac magnetic resonance imaging protocol using a 1.5 T MRI scanner (MAGNETOM Aera, Syngo Platform VD13A; Siemens Healthcare, Erlangen, Germany).^12^

#### Participant Classification and Outcomes

We used sex-specific thresholds to define abnormally low LVEF derived from within the same cohort, which were <48% for men and <51% for women, and classified participants as having normal or abnormal LVEF.^13^ We explored the relationship between CCI and laboratory and imaging biomarkers of systolic function, including LVEF and LV strain. The primary outcome was incident HF in those with normal LVEF at baseline.

#### Statistical Analyses

We used age- and sex-adjusted Cox regression models to determine the association between CCI and incident HF, excluding those who had an abnormal LVEF at baseline. Statistical analyses were done using R (version 4.1.1). All tests were 2-sided, and statistical significance was regarded as *P*<0.05. There was no imputation for missing data.

#### Ethical Considerations

UK Biobank received ethical approval from the NHS Research Ethics Service (11/NW/0382). We conducted this analysis under application 106965 Defining novel measures of LV contractility and their association with the risk of incident cardiovascular disease. All participants provided informed consent, and the study complied with the principles outlined in the Declaration of Helsinki.

## Results

### Baseline Characteristics of the NICE-CHF Cohort

After the exclusion of 182 patients who did not fulfil the diagnostic criteria for HF (Figure S1), and 72 for whom calculation of CCI was not possible (due to either insufficient endocardial definition or missing height, weight, or systolic blood pressure), the final data set consisted of 728 consecutively referred individuals who fulfilled the currently applied diagnostic criteria for HF.^1^ The mean age was 82.6±9.2 years, and 330 patients (45.3%) were male. The mean LVEF was 48.2±11.6% (range, 12.7–68.9%), 293 (40.2%) were classified as having HFrEF, and 435 (59.8%) as having HFpEF. The median CCI was 4.4 mm Hg/mL per m^2^ (range, 0.7–11.3 mm Hg/mL per m^2^).

### Association With LVEF

**Figure 1.**
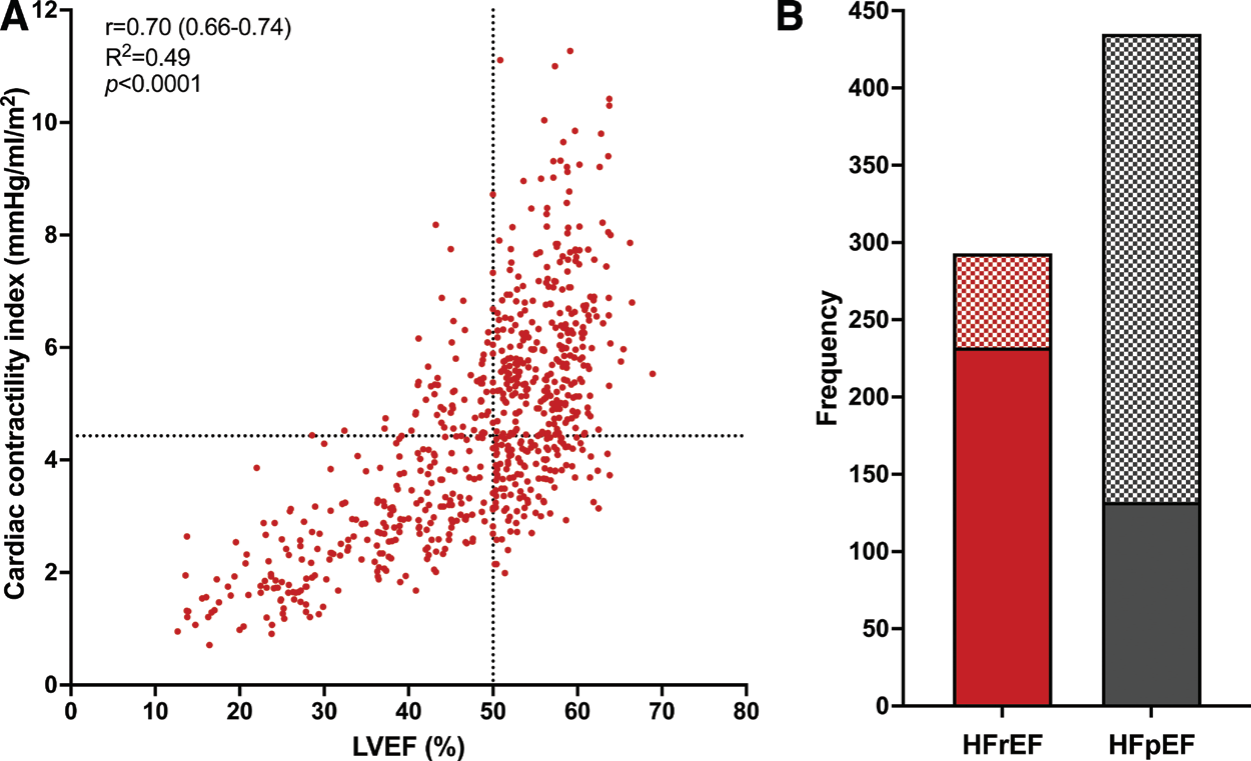
**The relationship between left ventricular ejection fraction (LVEF) and cardiac contractility index (CCI) in the NICE-CHF (National Institute for Clinical Excellence Guidelines on Chronic Heart Failure) cohort. A**, Scatter plots of LVEF and CCI and (**B**) bar charts showing the frequency of individuals with heart failure (HF) and reduced LVEF and low CCI (dark red), reduced LVEF and high CCI (light red), preserved LVEF and low CCI (dark gray) and preserved LVEF and high CCI (light gray). HFpEF indicates heart failure with preserved ejection fraction; HFrEF, heart failure with reduced ejection fraction; *r*, correlation coefficient; and *R*^2^; coefficient of determination.

LV end-systolic volume index and systolic blood pressure were minimally correlated (*r*=−0.11 [−0.18 to −0.037], *R*^2^ 0.012; *P*=0.003), suggesting their coupling may provide additional information regarding LV contractility than either parameter alone. Although there was a moderate, positive correlation between LVEF and CCI (*r*=0.70 [0.66–0.74], *R*^2^ 0.49; *P*<0.001), the latter was distributed widely for any given value of LVEF, especially for those with normal LVEF (Figure 1A). To explore the relationship between LVEF and CCI further, we divided participants by whether they had normal or abnormal LVEF, and by the median value of CCI (4.4 mm Hg/mL per m^2^), into 4 groups. Of those with HFrEF, 232 (79.2%) had low CCI, and 61 (20.8%) had high CCI. Of those newly diagnosed with HFpEF, 132 (30.3%) had low CCI, and 303 (69.7%) had high CCI (Figure 1B).

### Association With Natriuretic Peptides

We divided patients into tertiles of LVEF and CCI and found the median NTpro-BNP was 2119 (810–4827) pg/mL, 1003 (503–2113) pg/mL, and 655 (345–1344) pg/mL for tertiles 1, 2, and 3 of LVEF, respectively; and 2310 (933–5155) pg/mL, 921 (486–1759) pg/mL, and 715 (348–1362) pg/mL for tertiles 1, 2, and 3 of CCI, respectively (*P*<0.001 for trend in both comparisons; Figure S2). We also observed that the median NT-proBNP was higher in those with low CCI, regardless of whether they were classified as having HFrEF (2235 [788–5052] versus 813 [450–1810] pg/mL) or HFpEF (1153 [503–2353] pg/mL versus 761 [401–1409] pg/mL).

### Association With Clinical Characteristics

Those within the lowest tertiles of both LVEF and CCI were more often male, more frequently had ischemic heart disease and diabetes, and were less likely to have hypertension (Table 1). They also had, on average, worse conventional markers of disease severity, including lower systolic blood pressure, higher heart rate, and worse renal function. Despite this, the proportion of patients with New York Heart Association class III/IV symptoms was similar across tertiles of LVEF and CCI. Aside from the variables from which these groups were derived (systolic blood pressure and indexed LV volumes), the pattern of these associations was similar across tertiles whether patients were divided by LVEF or CCI. Within those classified as having HFpEF, those with low CCI were more often male, had ischemic heart disease, and had higher indexed left atrial volumes and tricuspid regurgitation velocity, implying higher filling pressures. They also more often had other markers of risk, including lower serum hemoglobin, worse renal function, and lower serum albumin compared with those with HFpEF and high contractility (Table 2).

**Table 1.**
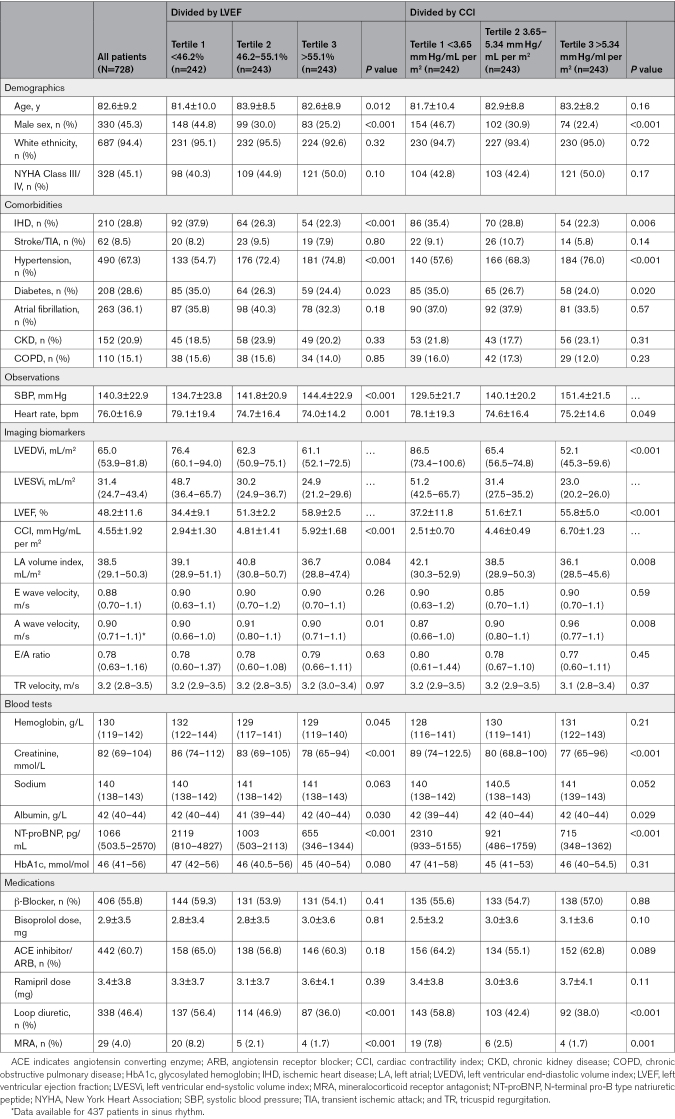
Clinical Characteristics of Patients in the NICE-CHF (National Institute for Clinical Excellence Guidelines on Chronic Heart Failure) Cohort Divided by Tertiles of LVEF and CCI

**Table 2.**
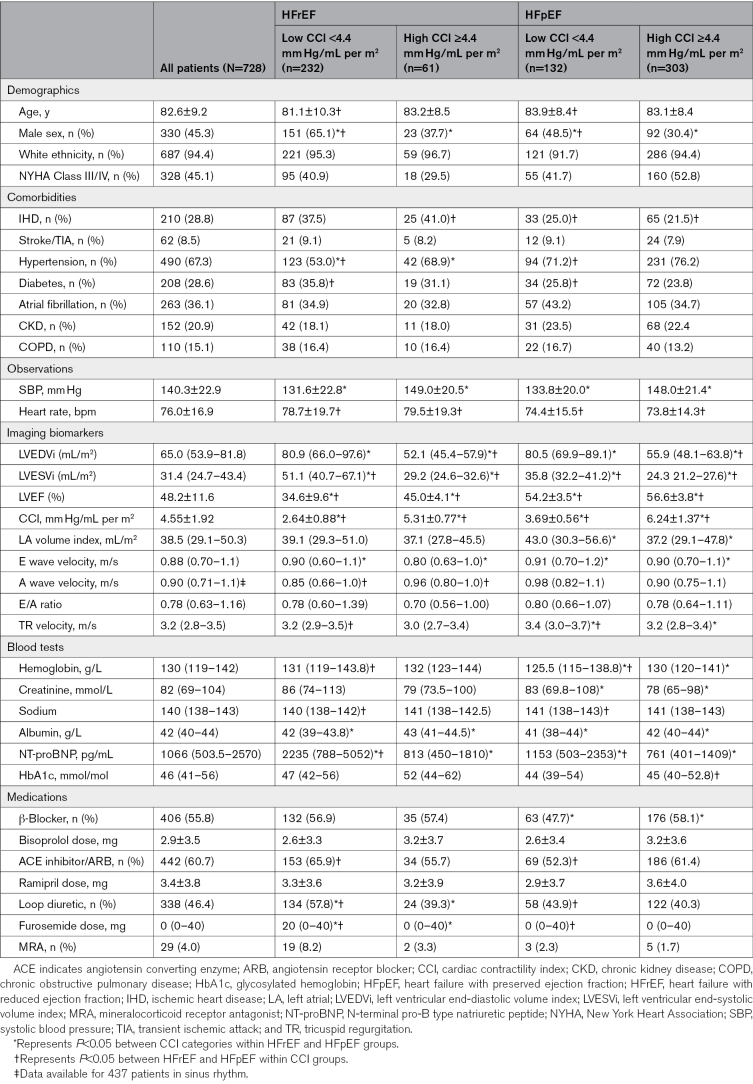
Clinical Characteristics of Patients in the NICE-CHF (National Institute for Clinical Excellence Guidelines on Chronic Heart Failure) With HFrEF and HFpEF Divided by Median CCI

### Association With Adverse Clinical Outcomes

**Figure 2.**
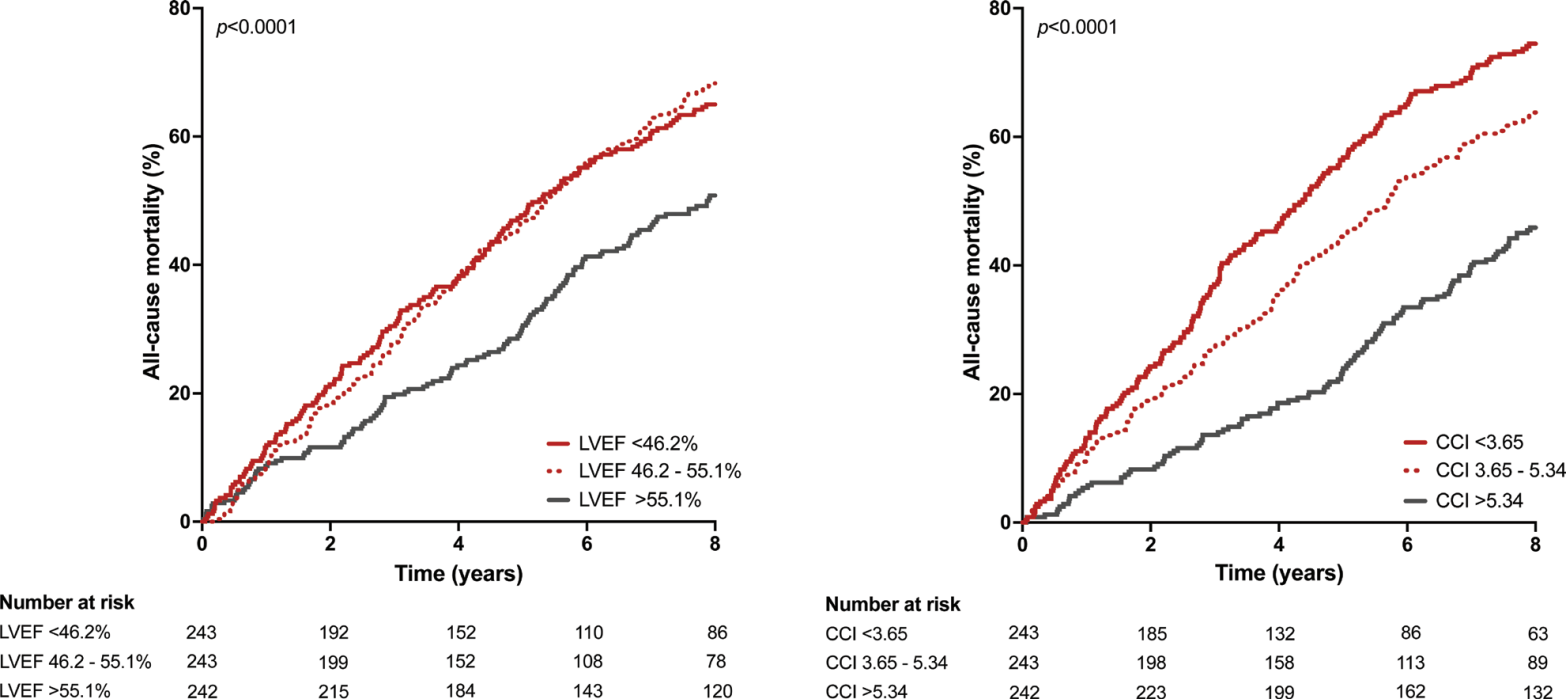
Kaplan-Meier plots of all-cause mortality divided by tertiles of left ventricular ejection fraction (LVEF) and cardiac contractility index (CCI) in the NICE-CHF (National Institute for Clinical Excellence Guidelines on Chronic Heart Failure) cohort.

During 4196 person-years of follow-up (median 5.9 [interquartile range, 2.9–9.0] years), a total of 491 (67.4%) people died. Of these, 151 (20.7%) were classified as cardiovascular deaths, 266 (36.5%) as noncardiovascular deaths and 74 (15.1%) were unascertained. We observed incrementally lower mortality risk across tertiles of CCI, whereas for LVEF, mortality risk was similar when comparing patients in tertiles 1 and 2 (Figure 2). The risk of all-cause mortality was more clearly distinguished by CCI than by LVEF regardless of whether divided into 2 groups, tertiles or quartiles (Figure S3).

**Figure 3.**
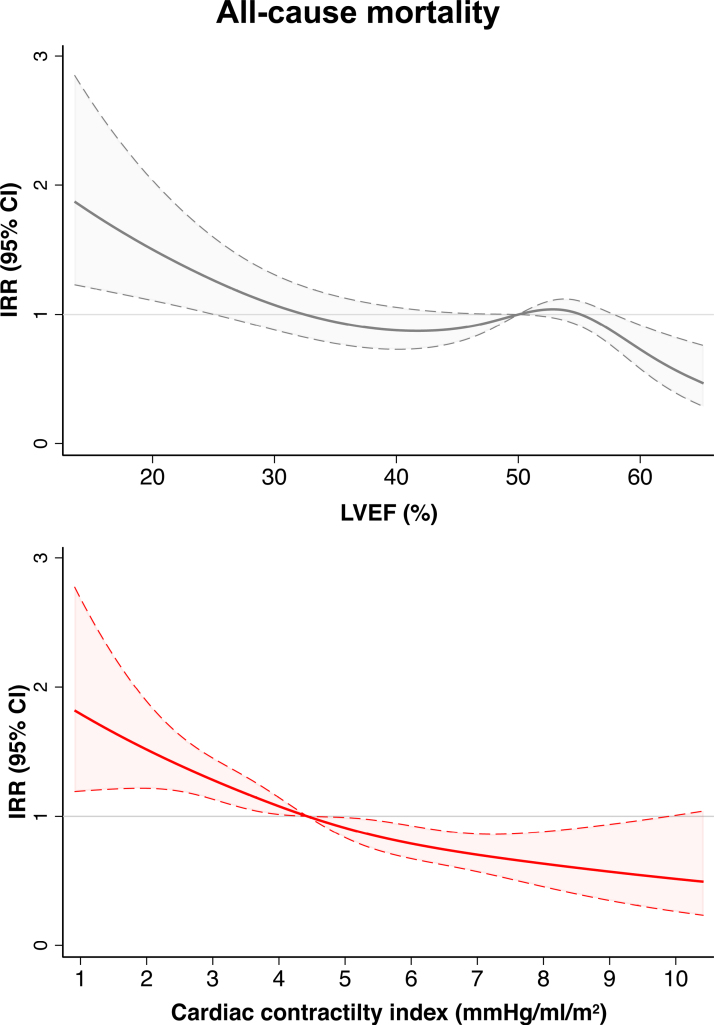
Restricted cubic splines displaying incidence rate ratios (IRR) and 95% CIs of all-cause mortality across left ventricular ejection fraction (LVEF) and cardiac contractility index (CCI) in the NICE-CHF (National Institute for Clinical Excellence Guidelines on Chronic Heart Failure) cohort.

We evaluated the relationship between CCI and LVEF, and all-cause mortality, cardiovascular mortality, and HF hospitalization risk by modeling each as continuous variables using restricted cubic splines. We observed a curvilinear relationship for both all-cause and cardiovascular mortality risk with CCI, with significantly higher or lower mortality rates across a broad range below or above the median, respectively. The relationship with LVEF was more complex, with no clear association with mortality rates across a wide range from 25% to 55% (Figure 3; Figure S4; Table S1). The relationship for both CCI and LVEF with HF hospitalization was curvilinear, with both CCI and LVEF clearly distinguishing risk throughout a broad range of values. We then included relevant covariates in an adjusted model, which were: age, male sex, ischemic heart disease, diabetes, hypertension, systolic blood pressure, heart rate, serum hemoglobin, creatinine, albumin, NT-proBNP, and left atrial volume index. The association between LVEF and all-cause mortality was no longer evident except for those with the highest LVEF, in whom the rate was lower (LVEF 60% IRR 0.83 [0.72–0.97], relative to LVEF 50%; Table 3). In contrast, the association with all-cause mortality remained evident for almost all specified values of CCI, even after adjusting for prognostically important covariates. The association between below median CCI and cardiovascular mortality remained evident in a competing risks regression model (subdistribution hazard ratio 0.65 [0.47–0.89]).

**Table 3.**
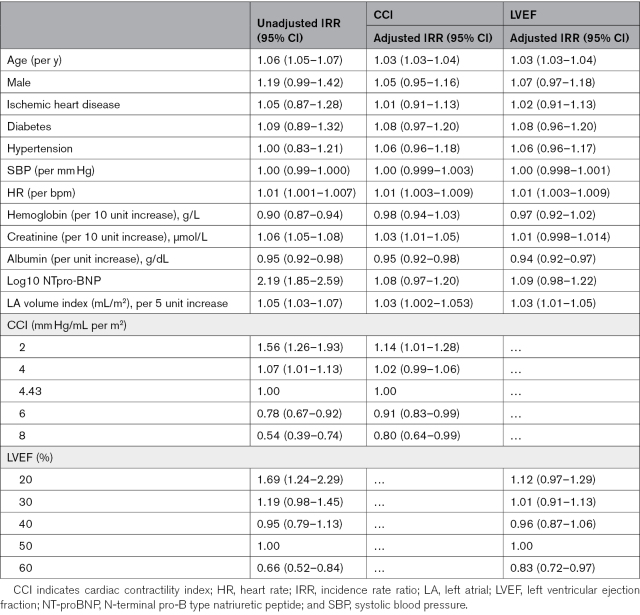
Poisson Regression Models Showing the Unadjusted Incidence Rate Ratios of All-Cause Mortality, As Well As Adjusted Multivariable Models With CCI and LVEF, Within the NICE-CHF (National Institute for Clinical Excellence Guidelines on Chronic Heart Failure) Cohort

**Figure 4.**
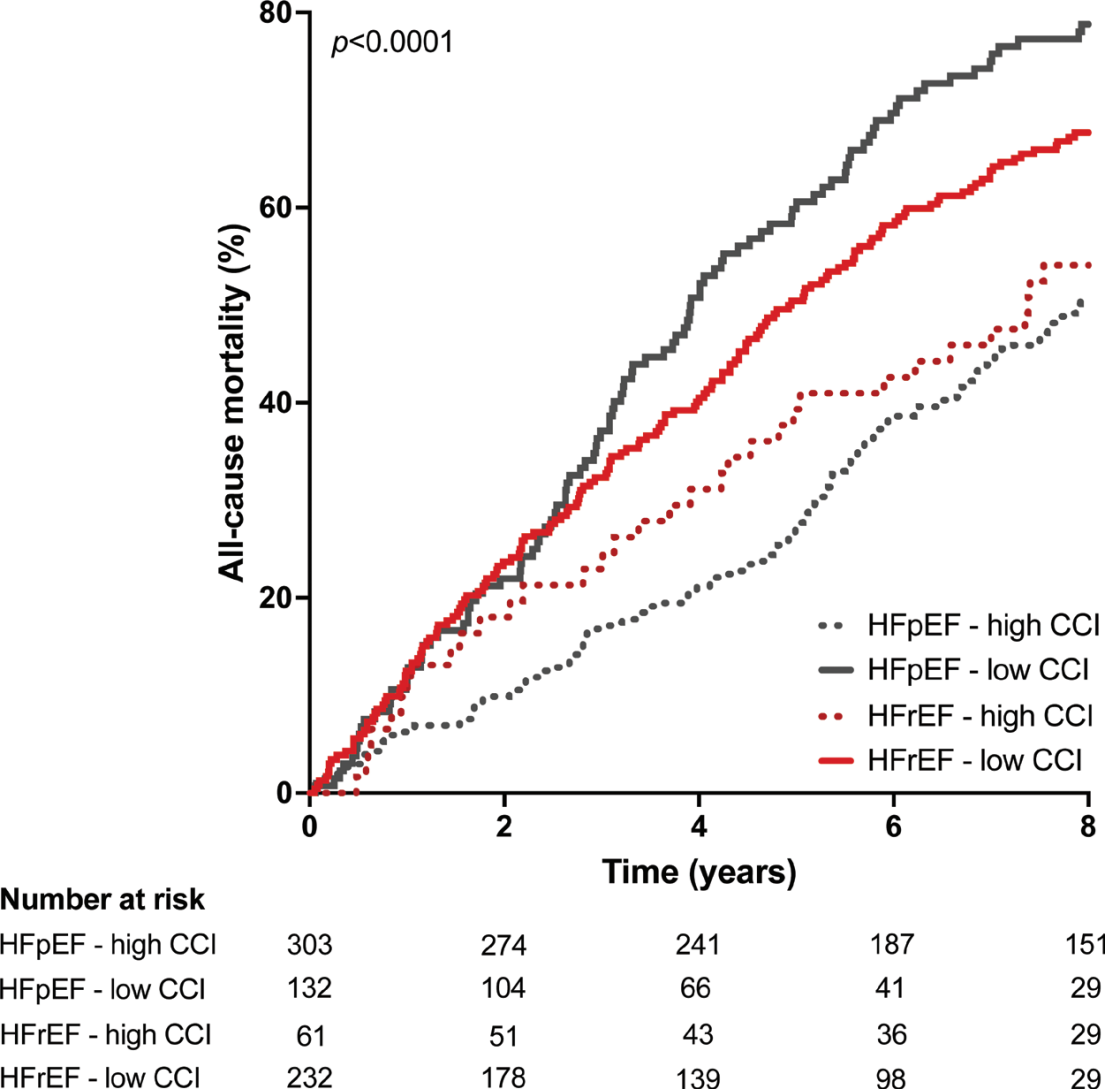
**Kaplan-Meier plot of all-cause mortality in patients with heart failure with reduced ejection fraction (HFrEF) and heart failure with preserved ejection fraction (HFpEF) who had cardiac contractility index (CCI) below or above the median value in the NICE-CHF (National Institute for Clinical Excellence Guidelines on Chronic Heart Failure) cohort.** Survival distributions between groups compared by Log-Rank test.

When LVEF and CCI were used in combination, we observed that the all-cause mortality risk was similar for people newly diagnosed with HFrEF, regardless of whether CCI was below or above the median value (observed event rate 14.2/100 patient years versus 10.6/100 patient years; *P*=0.096). However, for those newly diagnosed with HFpEF, below median CCI was associated with an all-cause mortality risk approximately twice that of those with above median CCI (observed event rate 17.3/100 patient years versus 8.8/100 patient years; *P*<0.001), similar to those with HFrEF (Figure 4).

### Analysis of CCI in UK Biobank Cohort

Initially, we included all participants who attended for the cardiac magnetic resonance imaging study after excluding 1168 with CCI or LVEF >3 standard deviations above or below the mean (to avoid extreme values), and 231 who were lost to follow-up. This resulted in 38 215 individuals who had a median age of 55 (49–61) years, of whom 47.8% were male (Table S2). The median LVEF was 56% (range, 26%–69%), and using sex-specific thresholds, 33 739 (88.3%) had a normal LVEF. The median CCI was 4.4 mm Hg/mL per m^2^ (range, 1.4–10.6), mirroring that noted in NICE-CHF.

We again observed a modest correlation between LVEF and CCI (*r*=0.53 [0.53–0.54], *R*^2^=0.29; *P*<0.001), with the latter distributed widely among those with normal LVEF (Figure S5A). Dividing participants into 4 groups according to LVEF and CCI, we observed similar proportions in the UK Biobank cohort. Of those with abnormal LVEF, 3702 (82.7%) had low CCI and 774 (17.3%) had high CCI. Of those with normal LVEF, 15 616 (46.3%) had low CCI and 18 123 (53.7%) had high CCI (Figure S5B).

**Figure 5.**
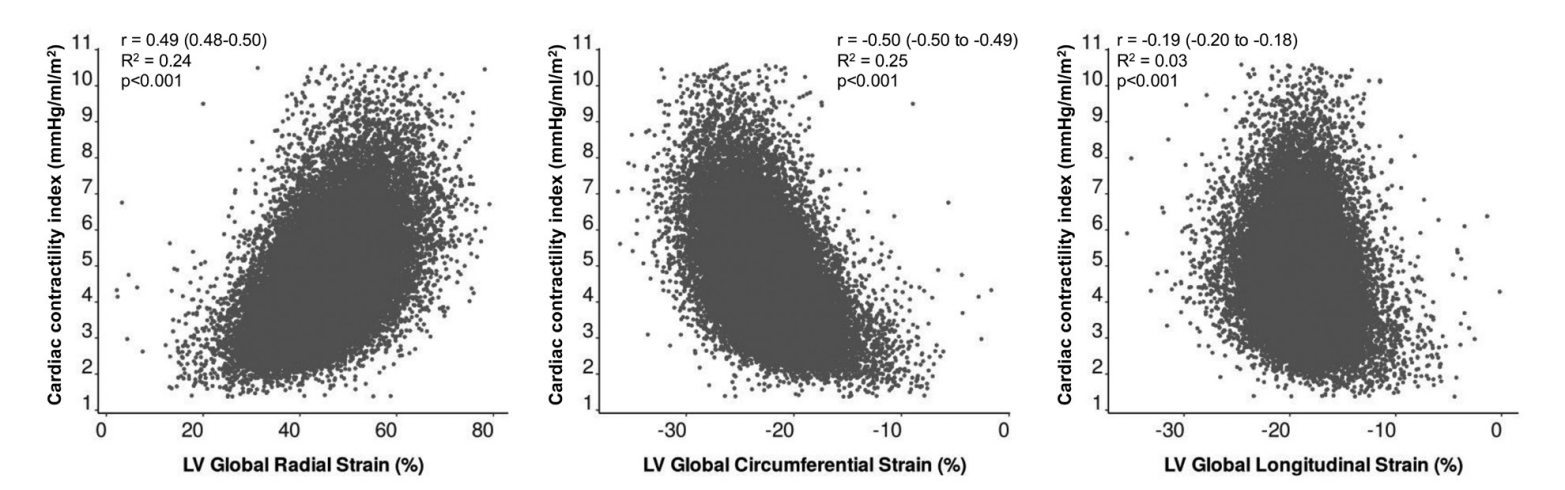
**Scatter plots of cardiac contractility index (CCI) compared to global radial, circumferential, and longitudinal strain in the UK Biobank cohort.** LV indicates left ventricular; *r*, correlation coefficient; and *R*^2^; coefficient of determination.

We observed that those with low CCI had more left atrial dilatation, more right ventricular dilatation, and worse right ventricular ejection fraction compared with those with high CCI. We examined the association between CCI and LV strain, an established imaging biomarker of LV contractility. We observed that CCI was moderately correlated with LV global circumferential (*r*=−0.50 [−0.50 to −0.49], *R*^2^=0.25; *P*<0.001) and radial strain (*r*=0.49 [0.48–0.50], *R*^2^=0.24; *P*<0.001), although was only weakly correlated with global longitudinal strain (*r*=−0.19 [−0.20 to −0.18], *R*^2^=0.03; *P*<0.001; Figure 5).

We then explored the association between CCI and the risk of incident HF, having excluded individuals with abnormal LVEF at baseline. During 195 584 person-years of follow-up (median, 5.5 [4.6–6.8] years), a total of 221 (0.7%) participants developed HF. In an age- and sex-adjusted Cox regression model, we observed that those with low CCI at baseline experienced an increased risk of future HF events (adjusted hazard ratio, 1.33 [1.01–1.75]; *P*=0.043; Table S3).

## Discussion

### Principal Findings

Our analysis of 2 complementary observational studies has several novel findings. First, we observed a broad range of LV contractility as determined by CCI, especially among those with a LVEF classified as normal. Second, we observed clear relationships between LV contractility and the risk of adverse clinical events in those with newly diagnosed HF, with the association with all-cause mortality remaining evident in a model including conventional markers of risk. Third, we found that when LV contractility was used to reclassify individuals with newly diagnosed HFpEF, we were able to identify a group with distinct clinical characteristics who were at increased risk of adverse clinical events. Fourth, we observed that impaired contractility (below median CCI) denoted increased risk of incident HF in people without HF or reduced LVEF at baseline. Notably, the median CCI was the same in the cohorts we studied, with below-median values being linked to incident HF in the healthy population and mortality in those with HF. Taken together, these data suggest that the assessment of LV contractility provides additional information beyond LVEF.

### LVEF: An Imperfect But Essential Tool in HF

First described over 6 decades ago,^14^ LVEF is a simple measure of LV systolic function that can be applied across imaging modalities. Much of current clinical practice is anchored to this biomarker, primarily because clinical trials supporting currently applied therapies enrolled participants who fell below arbitrary thresholds of LVEF and were perceived to be at the highest risk.^15,16^ As a consequence, for patients with HFrEF, 4 classes of medications targeting the neurohormonal maladaptations of the syndrome are proven to reduce hospitalizations and improve survival.^17^ However, with the possible exception of sodium-glucose cotransporter 2 inhibitors,^18^ the benefits of these agents are attenuated in those with higher LVEF,^19–21^ and for the overall population with HFpEF, no therapies have been shown to improve survival.^22,23^ As a consequence, LVEF persists as an imperfect but necessary tool for diagnosis, risk stratification, and defining the potential to benefit from evidence-based therapies.

### Limitations of LVEF

While LVEF remains central to our current understanding of HF, its relative simplicity comes with several well-known limitations, including poor intra and interobserver reproducibility, depending on the methods and experience of observers.^24^ Furthermore, while in the acute setting (eg, following myocardial infarction), a reduction in LVEF may truly reflect reduced LV contractility due to the loss of cardiomyocytes, subsequent LV dilatation means LVEF in HF is principally reflective of remodeling in response to loading conditions, and a poor reflection of myocardial contractile force.^25^ This might be acceptable in conditions such as dilated cardiomyopathy, in which increases in end-diastolic volume parallel reductions in systolic function. In this setting, where stroke volume is initially preserved, dilatation is reflected by a declining LVEF, which therefore provides a good approximation of systolic function.^25^ However, in other disease states such as restrictive or infiltrative cardiomyopathies, even when myocardial shortening is impaired, there is no resultant increase in LV volumes, such that the measured LVEF is normal, although systolic function is compromised. In our patients, we observed a broad range of LV contractility determined by CCI, especially evident among those with a LVEF classified as normal, both within a cohort with newly diagnosed HF and a healthy population without established HF at baseline. As such, where LVEF is normal, lower LV contractility can identify a subgroup who have a distinct phenotype, worse conventional markers of risk and increased risk of adverse clinical outcomes.

### Moving Beyond Ejection Fraction and Assessing LV Contractility

While LVEF is essential to guide care, additional indices of LV function may aid stratification of people with normal LVEF, with the potential to rationally guide the broader use of existing or emerging therapies. The current research landscape of alternative imaging biomarkers is dominated by speckle tracking techniques such as myocardial strain and strain rate. Myocardial strain has been shown to better detect subtle contractile dysfunction among patients with HFpEF,^26^ and global longitudinal strain is independently associated with adverse outcomes in both ambulatory and hospitalized patients with HFpEF, providing prognostic insights beyond LVEF.^3,27^ However, barriers exist to its more widespread implementation, such as variation between vendors, no currently agreed definition of what is normal, and limited data on the effects of loading conditions. In addition, the most commonly reported metric, global longitudinal strain, assesses systolic function in 1 plane meaning circumferential and radial dysfunction may be overlooked. In the UK Biobank cohort, we observed moderate correlations between CCI and global radial and circumferential strain. The correlation with global longitudinal strain was lower, which is consistent with its relationship to LVEF and likely reflects that both are dependent on the LV end-systolic volume.^26^

### Advantages of CCI Over LVEF

As originally described, CCI required the simultaneous measurement of LV volume and pressure at end-systole by invasive hemodynamic assessment. The use of systolic blood pressure obtained by a standard sphygmomanometer as a surrogate for end-systolic pressure has since been validated against invasive measurements,^7^ allowing for an entirely noninvasive assessment of the ratio between end-systolic volume and pressure. By incorporating systolic blood pressure, CCI is relatively load-independent, thereby better reflecting changes in LV contractility.^5^ It is unlikely that CCI entirely negates the effects of loading conditions, given that the end-systolic pressure differs from the systolic pressure, which is augmented by arterial stiffness. However, compared with more complex variables, it has the distinct advantage of requiring only routinely collected measurements, which should already be part of a minimum imaging data set and can be assessed across different imaging modalities.

### Simple Assessment of LV Contractility Provides Additional Prognostic Information

Previous studies have suggested that the relationship between peak systolic pressure and LV volume measured noninvasively is robustly associated with adverse outcomes, albeit in populations with a reduced LVEF.^28^ In our data, we show that CCI was associated with adverse clinical outcomes in an unselected population with HF, especially in those with HFpEF. The observation that CCI outperformed LVEF as a marker of risk in those with established HF is plausible, given that low systolic blood pressure is a marker of advanced HF, and also that this subgroup of patients seems to derive the greatest benefits from therapies which increase LV contractility.^29^

When applied to those with newly diagnosed HF, the relationship with all-cause and cardiovascular mortality risk was curvilinear for CCI and more robust than for LVEF, for which these associations were not evident throughout most of its range. A reduction in LV contractility probably does not make a diagnosis of HFpEF more likely, rather it identifies patients around one-third of patients who have worse conventional markers of risk, a distinct phenotype, and an increased risk of adverse clinical outcomes. Neither CCI nor LVEF was associated with New York Heart Association functional classification, which is known to poorly discriminate risk in HF populations.^30^ Dividing patients by the median value of CCI (4.4 mm Hg/mL per m^2^) was arbitrary, and although this value was near identical in the UK Biobank cohort, this is likely to be a chance finding. Nevertheless, by using applying this threshold, we were able to identify a phenotypically distinct subgroup of people with HFpEF who had worse outcomes, as well as predict future HF events in people without established HF at baseline.

### Strengths and Limitations

Our analysis includes individuals from 2 complementary observational cohort studies, the first of consecutively referred individuals who were newly diagnosed with HF according to currently applied definitions, and the second including a highly phenotyped cohort from the general population. Some limitations should be noted. First, NICE-CHF was an observational study conducted in a single center, which may limit generalizability, although the diverse characteristics of the area served by our center and the unique study design, which included all consecutively referred individuals to our service mitigates against this.^31^ Second, the lack of longitudinal imaging data means we cannot determine whether individuals with HFpEF went on to develop overt systolic dysfunction (LVEF <50%) in the future and whether CCI predicted this. Third, normal values of CCI have not been defined, although those derived from each data set were near identical, and by applying these thresholds we were able to identify individuals with a worse prognosis who had distinct characteristics. Fourth, we lack tissue-Doppler imaging meaning our characterization of diastolic function may be incomplete. Fifth, it is unclear the effect antihypertensive medications might have on CCI, how CCI might change over time in patients receiving medical therapy for HF, or whether these data are applicable to individuals with significant valvular heart disease given they were excluded from our data set. Sixth, the UK Biobank may not be representative of the general UK population with respect to socioeconomic deprivation, and ethnic minority groups, who were underrepresented.

### Conclusions

Our findings suggest that a simple measure of LV contractility offers additional prognostic information in people with HF and preserved LVEF, and also within the general population with normal LVEF. Identification of unappreciated contractile dysfunction in these circumstances may help better define risk and refine the phenotypic classification of these heterogenous populations. Future research should focus on post hoc analyses of published clinical trials testing proven HFrEF therapies in people with HFpEF, which have suggested negligible benefit. If these confirm that CCI can identify potential treatment responders, future randomized controlled trials could prospectively test the value of CCI in selective treatment responders, potentially leading to a new era in HFpEF care.

## Article Information

### Acknowledgments

This research was conducted using the UK Biobank Resource under Application Number 106965: Defining novel measures of left ventricular contractility and their association with the risk of incident cardiovascular disease. Full details of its design and conduct are available online (https://www.ukbiobank.ac.uk). This work uses data provided by patients and collected by the National Health Service as part of their care and support; Copyright © (2022), NHS Digital; reused with the permission of UK Biobank. All rights reserved. This research used data assets made available by the National Safe Haven as part of the Data and Connectivity National Core Study, led by Health Data Research UK in partnership with the Office for National Statistics, and funded by UK Research and Innovation (research which commenced between October 1, 2020, and March 31, 2021; grant ref MC_PC_20029; and from April 1, 2021 to September 30, 2022; grant ref MC_PC_20058). The graphical abstract was created using BioRender. C.A. Cole, Drs Lowry, Paton, Slater, Cubbon, Kearney, Witte, and Gierula collected the data. Drs Straw, Brown, Relton, MH, and Cubbon analyzed the data. Dr Straw produced the first draft of the article. All other authors provided critical revision.

### Sources of Funding

This study is supported by a British Heart Foundation Clinical Research Training Fellowship awarded to Dr Sam Straw (FS/CRTF/20/24071). Drs Straw, Drozd, Gierula, and Paton are supported by the National Institute for Health and Care Research. Drs Conning-Rowland, Lowry, Cubbon, and Kearney are supported by the British Heart Foundation. Dr Levelt is supported by the Wellcome Trust. This is independent research funded by the British Heart Foundation and performed at the National Institute for Health and Care Research Leeds Biomedical Research Centre (BRC; NIHR203331). The views expressed are those of the authors and not necessarily those of the British Heart Foundation, the National Institute for Health and Care Research, or the Department of Health and Social Care. The authors acknowledge the support of the National Institute for Health and Care Research Leeds Cardiovascular Clinical Research Facility.

### Disclosures

Dr Straw has received speaker fees, honoraria, and a research grant from AstraZeneca. Dr White has received personal fees from Medtronic, Cardiac Dimensions, Novartis, Abbott, BMS, Pfizer, and Bayer, and has received an unconditional research grant from Medtronic. Dr Kearney has received personal fees and a research grant from AstraZeneca. Dr Gierula has received personal fees from Abbott, Medtronic, and MicroPort, and has received an unrestricted research grant from Medtronic. The other authors report no conflicts.

### Supplemental Material

Supplementary Methods

Supplementary Results

Tables S1–S3

Figures S1–S5

References 32–34

## Supplementary Material
