# Supplementary material for: Assessing LV Contractility Identifies Populations With Preserved Ejection Fraction at Risk of Adverse Heart Failure Outcomes
Source: Circ Cardiovasc Imaging. 2025 Jul 16;18(9):e018370. doi: 10.1161/CIRCIMAGING.125.018370 (PMC7617944; doi:10.1161/CIRCIMAGING.125.018370)
Supplement: Supplementary file 1 [file hci-18-e018370-s001.pdf]

# SUPPLEMENTARY MATERIAL

**SUPPLEMENTARY METHODS**

NICE-CHF cohort

*Study population*

Patients were evaluated in a secondary care specialist heart failure clinic. Upon arrival, demographic details, medical history, and currently prescribed medications were recorded. We defined ischaemic heart disease as any of prior myocardial infarction, prior percutaneous coronary intervention or coronary artery bypass grafting, or invasive angiography or other anatomical or functional test suggesting significant coronary artery disease. Height and weight were measured, and a venous blood sample was taken and tested for full blood count, electrolytes, and assessment of renal and liver function. NT-proBNP had previously been measured at the point of referral using samples collected in primary care, which were analysed at our institution using the Immulite 2000 assay (Siemens Healthcare Diagnostics, Camberley, UK) which has an inter-batch coefficient variation of 8.9% at 350pg/mL and 5.9% at 4100pg/ml. A standard 12-lead electrocardiogram was recorded at 25mm/s and two-dimensional transthoracic echocardiography was performed.

*Imaging acquisition and analysis*

Transthoracic echocardiographic images were obtained by senior cardiac sonographers, who obtained a minimum dataset according to recommendations of the American Society of Echocardiography and European Association of Cardiovascular Imaging.[32] Patients were scanned semi-supine, with standard views obtained to exclude significant valvular heat disease or alternative diagnoses (Supplementary Table 1). Images were then sent to digital storage media and, for the present analysis were analysed offline using Medcon (McKesson Cardiology, Irving TX, USA) by two senior accredited cardiac sonographers (CC, JG) who were blinded to patient characteristics and measurements of NT-proBNP.

*Systolic indices*

Where endocardial border definition allowed, LV end-diastolic volume, LV end-systolic volume end-systole were measured in apical two and four-chamber views using the biplane method of disks. Sonographers were advised to use the frame at which the LV cavity was largest and smallest, to define end-diastole and end-systole, respectively. For patients in sinus rhythm, we determined LV volumes during a single cardiac cycle,

and for patients in atrial fibrillation an average was taken of three cardiac cycles. To determine CCI, systolic blood pressure was used as a surrogate of LV end-systolic pressure as previously described,[7] and calculated by dividing systolic blood pressure by LV end-systolic volume indexed to body surface area. All measurements were indexed for body surface area, which was calculated using the Mosteller equation.[33]

*Diastolic indices*

Where endocardial border definition allowed, left atrial volume at end-systole was measured in apical two and four-chamber views using the biplane method of disks. Sonographers were advised to use the frame at which the LV cavity was smallest to define end-systole. Left atrial volume was indexed for body surface area, which was calculated using the Mostellar equation.[33] Peak mitral valve inflow velocities were measured in apical four-chamber views. The peak E wave velocity was measured in early diastole, and the peak A wave velocity was measured in late diastole for patients in sinus rhythm. The ratio between E and A waves was calculated for patients in sinus rhythm. Peak tricuspid regurgitation velocity was measured in a modified apical four-chamber view, for patients with at least mild tricuspid regurgitation.

*Participant classification*

We classified patients according to the current European Society of Cardiology guidelines.[1] We required participants to have signs and/or symptoms of CHF, elevated natriuretic peptides (N-terminal B-type natriuretic peptide ≥125pg/mL), and either LVEF <50%, or LVEF ≥50% as well as relevant structural heart disease (either left atrial dilatation, LV hypertrophy) or diastolic dysfunction. Patients without these echocardiographic features, including those in whom symptoms were attributable to significant valvular disease were excluded. We divided patients into tertiles based on measures of LV function, to determine the clinical characteristics and outcomes of these groups. The ranges for tertiles one, two and three of LVEF were <46.2%, 46.2-55.1% and >55.1%; the ranges for tertiles one, two and three of CCI were <3.65mmHg/ml/$m^2$, 3.65-5.34mmHg/ml/$m^2$ and >5.34mmHg/ml/$m^2$. In both cohorts, we subdivided participants into four groups according to thresholds of LVEF and CCI. We used the median value of CCI, which was 4.4mmHg/ml/$m^2$ in both datasets, for LVEF we divided patients as to whether they had LVEF <50% or ≥50%, and also did

sensitivity analyses divided by whether they had HFrEF, heart failure with mildly reduced ejection fraction (HFmrEF), or HFpEF.

*Ascertainment of outcomes*

Outcomes data were collected using linked Hospital Episode Statistics and Office of National Statistical mortality data with final censorship in January 2022. We extracted the underlying (primary) cause of death as stipulated on the individual's death certificate obtained from the electronic healthcare record or by request to the coroner, coded according to the ICD-10 from death certification data, classified as cardiovascular (I00-I99, excluding infection codes) or non-cardiovascular. We recorded hospitalisation data obtained from the electronic healthcare record and classified these as relating to cardiovascular or non-cardiovascular causes according to the documented primary reason for hospitalisation.

*Statistical analyses*

NICE-CHF included consecutively referred patients over the course of one year, and therefore the sample size was not prespecified. The interobserver variability for LV end-diastolic and end-systolic volumes were compared in a random sample of 10% of patients assessed by both observers by intra-class correlation coefficient. For patients in sinus rhythm these were 0.99 (95% CI 0.98-0.99) and 0.99 (95% CI 0.98-0.99), and for patients in atrial fibrillation these were 0.98 (95% CI 0.96-0.99) and 0.98 (95% CI 0.97-0.99), for LV end-diastolic and end-systolic volumes, respectively.

Normality of distribution was confirmed using skewness tests. Unless otherwise stated, continuous variables are presented as mean ± standard deviation where normally distributed, or as median ($25^{th}$ and $75^{th}$ centiles) if non-normally distributed, with discrete variables presented as number (percentage). Groups were compared using t-tests or one-way analysis of covariance for normally distributed continuous data, Mann-Whitey or Kruskal-Wallis H tests for non-normally distributed data, and Pearson $\chi^2$ test for categorical variables.

Scatter plots were constructed for CCI compared to LVEF with correlation determined by Pearson's correlation coefficients (r) and coefficient of determination ($R^2$). We

plotted Kaplan Meier curves to illustrate all-cause mortality rates, with significance testing between groups determined by log-rank test.

We found the proportional hazards assumptions were not valid for LVEF and CCI, we therefore estimated incident rate ratios (IRR) for adverse clinical outcomes using Poisson regression models. Exposure time was modelled, but time-varying covariates were not used. Models including cubic splines with three, four or five knots and first and degree fractional polynomials were compared by Akaike and Bayesian information criterion scores. For LVEF we chose four, four, and three knots for all-cause mortality, cardiovascular mortality and heart failure hospitalisation, respectively; for CCI we chose four, three, and three knots for all-cause mortality, cardiovascular mortality, and heart failure hospitalisation, respectively. IRRs and their 95% confidence intervals (CI) estimated for LVEF and CCI pertain to specific points (LVEF 20, 30, 40, and 60% compared with 50%, and CCI 2, 4, 6 and 8mmHg/ml/$m^2$ compared with 4.4mmHg/ml/$m^2$ which was the median value). Covariates included in a multivariable Poisson regression model were age, sex, ischaemic heart disease, diabetes mellitus, hypertension, systolic blood pressure, heart rate, haemoglobin, creatinine, albumin and NT-proBNP, in which non-normally distributed continuous data were $\log_{10}$ transformed.

Statistical analyses were done using Stata/MP (version 16.1, StataCorp LLC, College Station, TX, USA) and R (version 4.1.1), with figures illustrated using PRISM (version 9, GraphPad Software Inc, San Diego, CA). All tests were two-sided and statistical significance was regarded as $p<0.05$. There was no imputation for missing data.

UK Biobank cohort

*Study population*

Full details of the design and conduct of UKB are available online (https://www.ukbiobank.ac.uk). At study recruitment, participants underwent comprehensive baseline assessment with a touchscreen questionnaire and nurse-led interview to capture sociodemographic characteristics, comorbidities, currently prescribed medications, in addition to physical measures and blood testing.[34] From 2014, all surviving participants were invited by email and postal mail to take part in a multimodality imaging assessment, including cardiac magnetic resonance (CMR)

imaging. At the time of analysis, data were available for approximately 40,000 participants who underwent CMR imaging.

*Imaging acquisition and analysis*

Participants underwent a standardised 20-minute CMR protocol using a 1.5 Tesla MRI scanner (MAGNETOM Aera, Syngo Platform VD13A, Siemens Healthcare, Erlangen, Germany) at four sites in the UK. Full details of the CMR image acquisition have been previously published.[12] The protocol included bright blood anatomical assessment, cine imaging, myocardial tagging, native T1 mapping, aortic flow, and imaging of the thoracic aorta. LV chamber volumes, and LVEF were derived from the short-axis cine stack, and tagging sequences were used for the measurement of LV regional and global radial, circumferential and longitudinal strain.

Manual analysis at two core laboratories (London and Oxford) of all four chambers was completed for the first 5000 CMR imaging datasets according to a pre-defined protocol, by readers who had received dedicated training and standardized quality control. A fully automated image analysis tool was developed to analyse imaging datasets, validated against the 5000-participant reference cohort.

*Participant classification*

In UKB we used sex-specific thresholds derived from within the same dataset, which were < or ≥48% for men, and < or ≥51% for women, as previously published.[35]

*Ascertainment of outcomes*

Incident heart failure events in the UKB cohort were identified from linked digital registries including Hospital Episode Statistics England (HES), Scottish Morbidity Record (SMR), Patient Episode Database and primary care linked records. Self-reported heart failure codes were excluded to focus solely on evens confirmed in medical records. The time to heart failure was defined as the interval between the date of imaging assessment centre visit and the date that heart failure was first reported. For time to event analyses, we excluded participants with abnormal cardiac function at baseline, using sex specific cut-offs for LVEF, as our aim was to capture incident heart failure events in those with normal LVEF at baseline.

*Statistical analyses*

From 2014, all surviving participants were approached by email and by post to take part in a multimodality imaging assessment, with the aim of imaging 20% of the original cohort.[1] Normality of distribution was confirmed using skewness tests. Unless otherwise stated, continuous variables are presented as median (25th and 75th centiles), with discrete variables presented as number (percentage). Groups were compared using t-tests or one-way analysis of covariance for normally distributed continuous data, Mann-Whitey or Kruskal-Wallis H tests for non-normally distributed data, and Pearson $\chi^2$ test for categorical variables.

We excluded participants with outlying CCI or LVEF values which were ≥3 standard deviations above or below the mean. Scatter plots were constructed for CCI compared to NT-proBNP, LVEF, longitudinal, radial and circumferential strain, with correlation determined by Pearson's correlation coefficients (r) and coefficient of determination ($R^2$). Time to event analyses were done using the R package "survival" (version 3.2.13). We found that the proportional hazards assumptions were valid for LVEF and CCI, and therefore estimated the risk of incident heart failure using age and sex adjusted Cox proportional hazard ratios (HR) and their 95% CI. Exposure time was modelled, but time-varying covariates were not used.

Statistical analyses were done using R (version 4.1.1). All tests were two-sided and statistical significance was regarded as $p<0.05$. There was no imputation for missing data.

## SUPPLEMENTARY RESULTS

<u>Sensitivity analyses according to classification of heart failure</u>

Within those with abnormal LVEF (<50%) below median CCI was not associated with an increased risk of all-cause or cardiovascular mortality, or heart failure hospitalisation ($p>0.05$ for all comparisons) regardless of whether individuals had HFrEF (LVEF <40%) or HFmrEF (40-49%).

**SUPPLEMENTARY FIGURES**

Figure S1

Title: Flow diagram showing the reasons for exclusion in the NICE-CHF cohort.

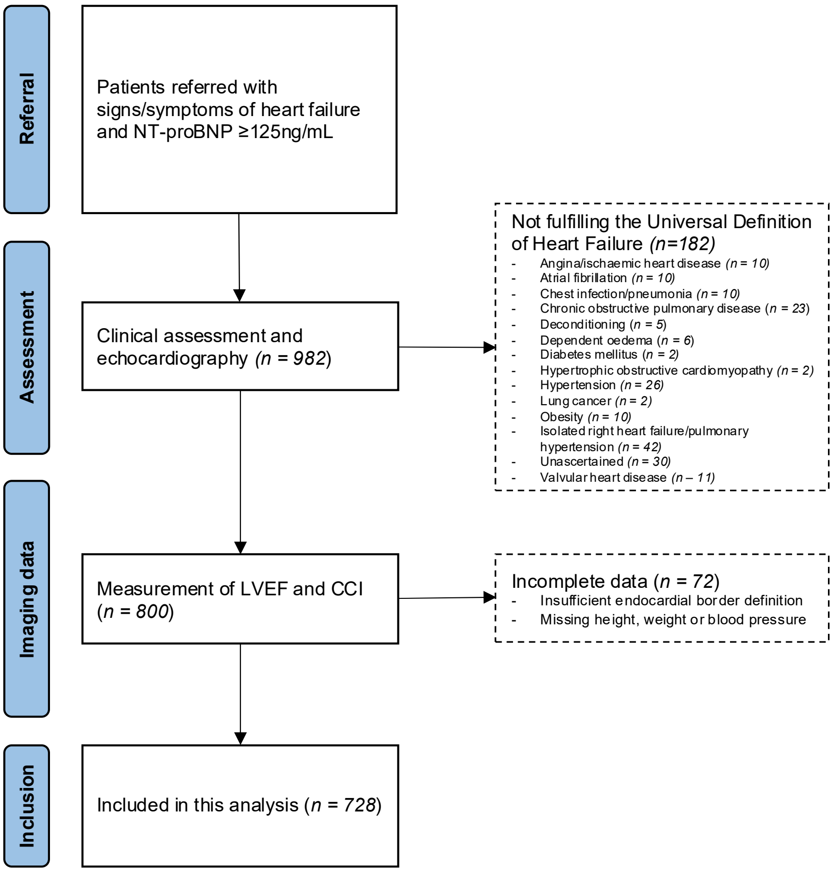


NT-proBNP; N-terminal B-type natriuretic peptide, LVEF; left ventricular ejection fraction, CCI; cardiac contractility index.

Figure S2

Title: Bar charts showing levels of NT-proBNP between groups in the NICE-CHF cohort.

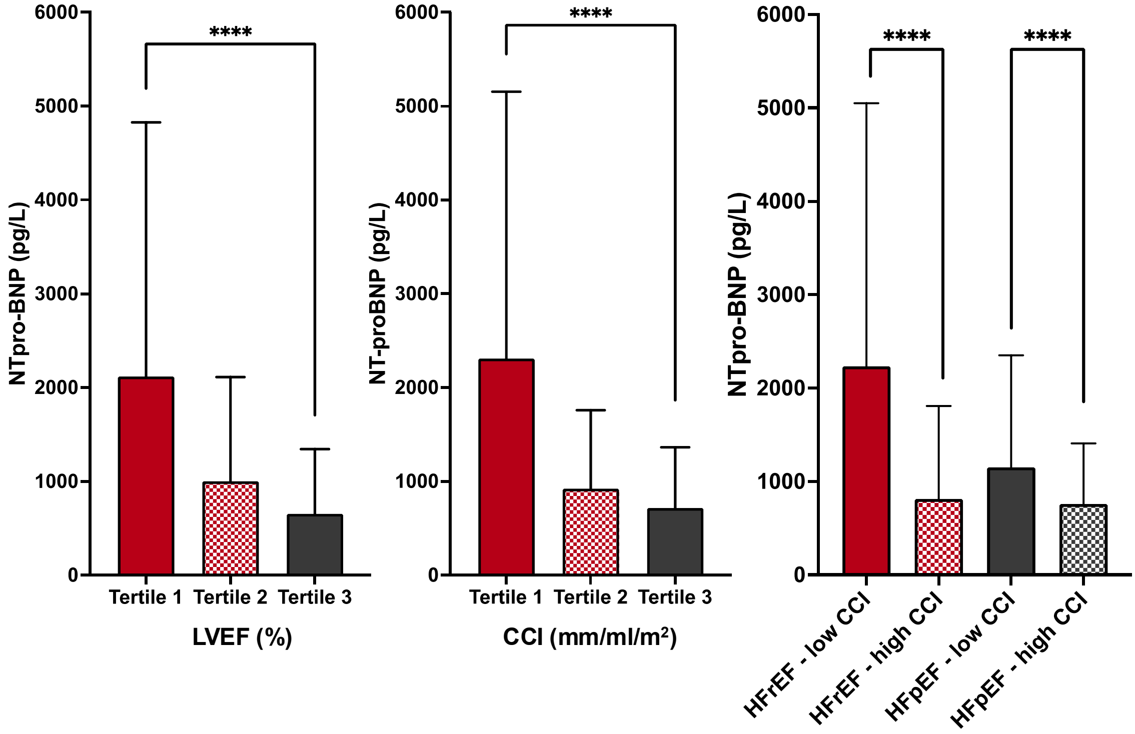


NT-proBNP; N-terminal B-type natriuretic peptide, LVEF; left ventricular ejection fraction, CCI; cardiac contractility index, HFrEF; heart failure with reduced ejection fraction, HFpEF; heart failure with preserved ejection fraction.

Figure S3

Title: Kaplan-Meier plots of all-cause mortality divided by median values or into quartiles of LVEF and CCI in the NICE-CHF cohort.

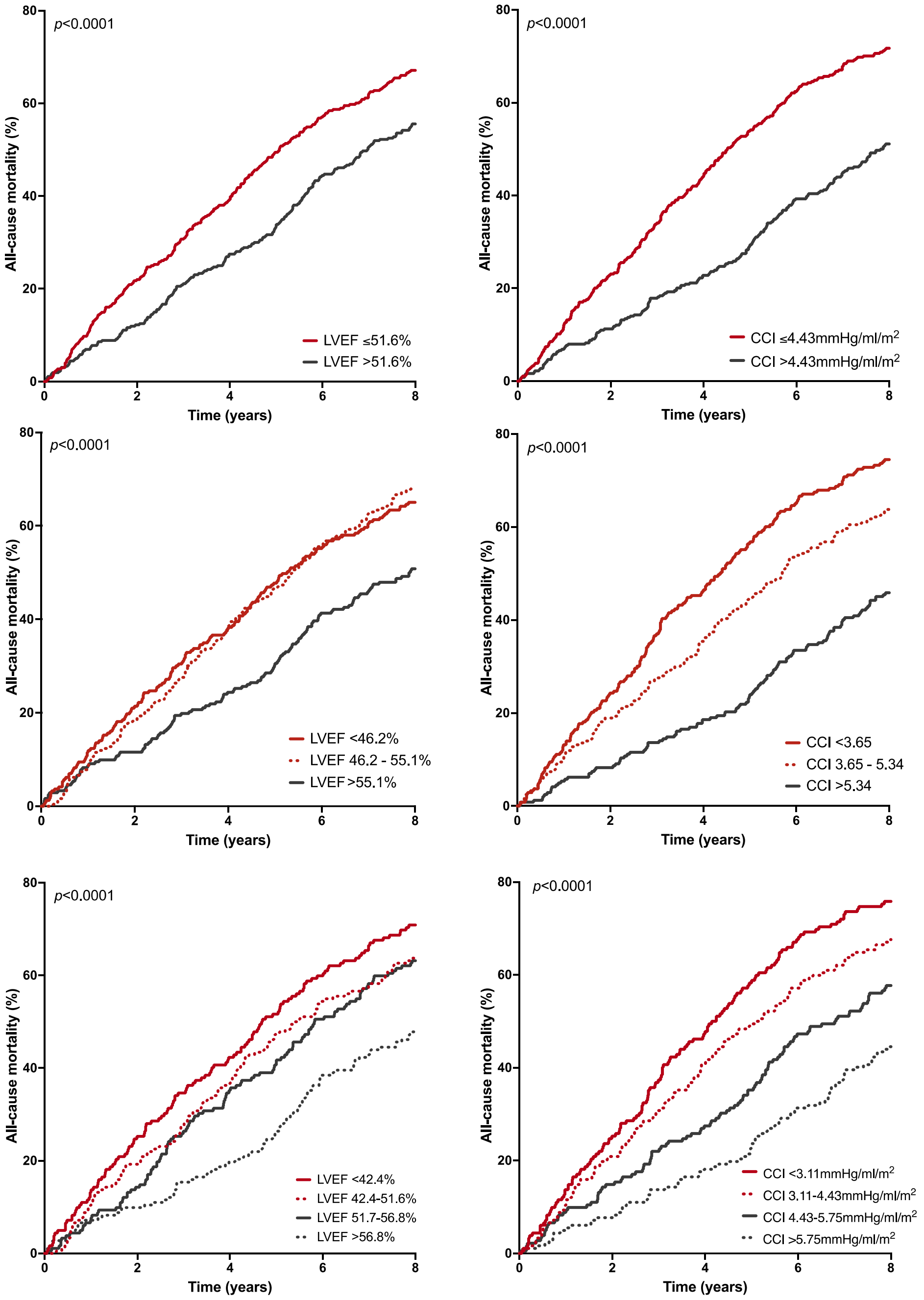


LVEF; left ventricular ejection fraction, CCI; cardiac contractility index.

Figure S4

Title: Restricted cubic splines of the association between CCI and LVEF with cardiovascular mortality risk, and heart failure hospitalisation risk in the NICE-CHF cohort.

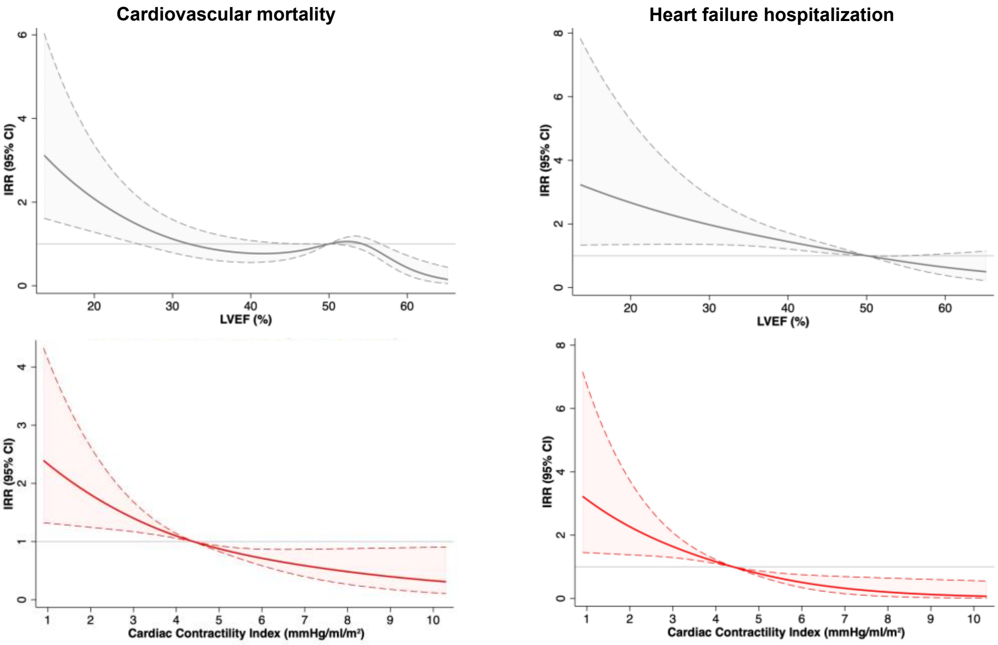


IRR; incidence rate ratio, LVEF; left ventricular ejection fraction.

<u>Figure S5</u>

Title: The relationship between LVEF and CCI in UK Biobank. (A) Scatter plots of LVEF and CCI and (B) bar charts showing the frequency of individuals with abnormal LVEF and low CCI (dark red), abnormal LVEF and high CCI (light red), normal LVEF and low CCI (dark grey) and normal LVEF and high CCI (light grey).

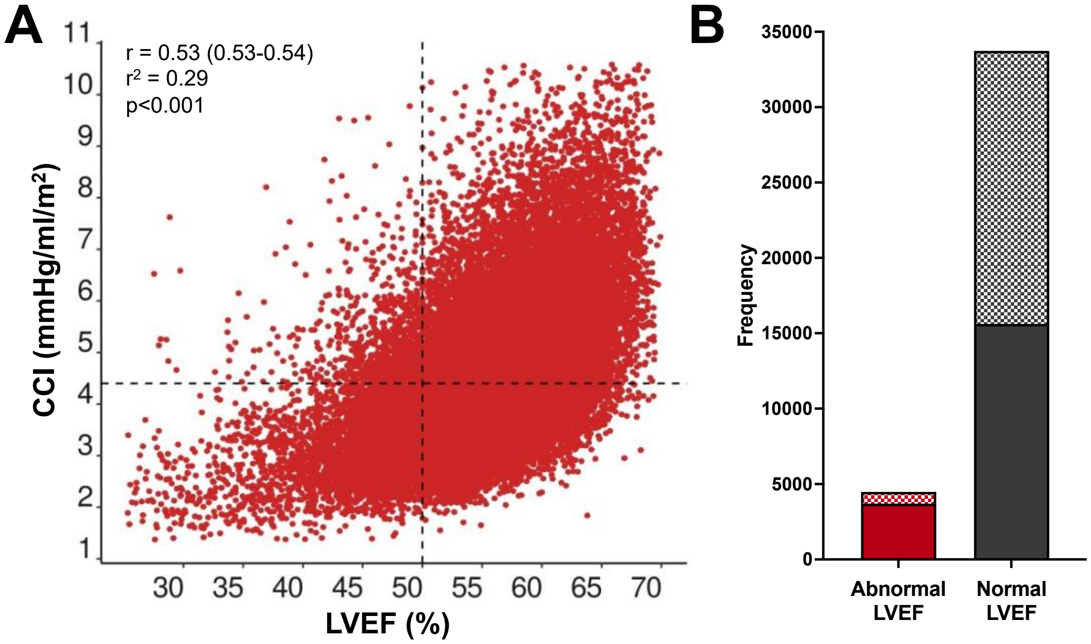


r; correlation coefficient, R2; coefficient of determination, CCI; cardiac contractility index, LVEF; left ventricular ejection fraction.

**SUPPLEMENTARY TABLES**

Table S1

Table displaying incident rate ratios pertaining to specific values of LVEF and CCI for the risk of cardiovascular death and heart failure hospitalization, determined using unadjusted Poisson regression models within the NICE-CHF cohort.

| | **Cardiovascular death Unadjusted IRR (95%CI)** | **HF hospitalization Unadjusted IRR (95%CI)** |
|---|---|---|
| CCI (mmHg/ml/m$^2$) | | |
| 2 | 1.82 (1.25-2.65) | 2.33 (1.44-3.78) |
| 4 | 1.10 (1.06-1.15) | 1.17 (1.11-1.24) |
| 4.4 | 1.00 | 1.00 |
| 6 | 0.72 (0.60-0.87) | 0.53 (0.36-0.76) |
| 8 | 0.49 (0.27-0.88) | 0.22 (0.07-0.66) |
| LVEF (%) | | |
| 20 | 2.08 (1.28-3.37) | 2.84 (1.47-5.46) |
| 30 | 1.12 (0.79-1.59) | 2.03 (1.41-2.93) |
| 40 | 0.78 (0.56-1.08) | 1.45 (1.22-1.72) |
| 50 | 1.00 | 1.00 |
| 60 | 0.43 (0.26-0.73) | 0.66 (0.40-1.09) |

IRR; incidence rate ratio, CI; confidence interval, HF; heart failure, CCI; cardiac contractility index, LVEF; left ventricular ejection fraction.

## Table S2

Clinical characteristics of participants in the UK Biobank cohort divided by normal or abnormal LVEF (≥ or <48% for men, and ≥ or <51% for women) and by median CCI.

| | Missing | Abnormal LVEF | | *p*-value | Normal LVEF | | *p*-value |
|---|---|---|---|---|---|---|---|
| | | **Low CCI** (n=3,702) | **High CCI** (n=774) | | **Low CCI** (n=15,616) | **High CCI** (n=18,123) | |
| ***Demographics*** | | | | | | | |
| Age (years) | 0 | 57 (50-62) | 60 (56-64) | <0.001 | 52 (46-59) | 57 (51-62) | <0.001 |
| Male sex (n[%]) | 0 | 1949 (52.7) | 269 (34.8) | <0.001 | 8 834 (56.6) | 7 232 (39.9) | <0.001 |
| White ethnicity (n[%]) | 109 | 3622 (97.8) | 748 (96.6) | 0.055 | 15 200 (97.3) | 17 450 (96.3) | <0.001 |
| Smoking | 92 | | | | | | |
| Never (n[%]) | - | 2 189 (59.1) | 451 (58.3) | 0.289 | 9 676 (62.0) | 10 883 (60.1) | <0.001 |
| Former (n[%]) | - | 1201 (32.4) | 270 (34.9) | 0.289 | 4903 (31.4) | 6185 (34.1) | <0.001 |
| Current (n[%]) | - | 299 (8.1) | 52 (6.7) | 0.289 | 1003 (6.4) | 1011 (5.6) | <0.001 |
| BMI (kg/m$^2$) | 52 | 25.7 (23.3-28.4) | 27.0 (24.3-30.2) | <0.001 | 25.4 (23.2-27.9) | 26.5 (24.1-29.4) | <0.001 |
| Townsend deprivation index | 39 | | | | | | |
| Q1 (n[%]) | - | 728 (19.7) | 142 (18.4) | 0.383 | 3123 (20.0) | 3652 (20.2) | 0.947 |
| Q1 (n[%]) | - | 772 (20.9) | 145 (18.7) | | 3105 (19.9) | 3617 (20.0) | |
| Q3 (n[%]) | - | 758 (20.5) | 159 (20.5) | | 3114 (19.9) | 3615 (20.0) | |
| Q4 (n[%]) | - | 681 (18.4) | 162 (20.9) | | 3171 (20.3) | 3619 (20.0) | |
| Q5 (n[%]) | - | 759 (20.5) | 166 (21.5) | | 3089 (19.8) | 3599 (19.9) | |
| ***Observations*** | | | | | | | |
| SBP (mmHg) | 0 | 132 (121-145) | 143 (130-155) | <0.001 | 130 (119-141) | 141 (129-153) | <0.001 |
| Heart rate (beats/min) | 0 | 63 (56-70) | 70 (63-78) | <0.001 | 58 (53-64) | 64 (58-71) | <0.001 |
| ***Imaging biomarkers*** | | | | | | | |
| LVEDVi (ml/m$^2$) | 0 | 77 (69-89) | 56 (50-62) | <0.001 | 80 (73-88) | 65 (58-72) | <0.001 |
| LVESVi (ml/m$^2$) | 0 | 42 (37-49) | 30 (26-33) | <0.001 | 36 (31-40) | 27 (24-30) | <0.001 |
| CCI (mmHg/ml/m$^2$) | 0 | 3.2 (2.7-3.7) | 5.0 (4.6-5.6) | <0.001 | 3.7 (3.3-4.1) | 5.4 (4.8-6.2) | <0.001 |
| LVEF (%) | 0 | 46 (42-47) | 47 (45-49) | <0.001 | 55 (52-58) | 59 (56-62) | <0.001 |
| Global Longitudinal strain (%) | 1985 | -16.4 (-18.2 to -14.5) | -16.8 (-18.7 to -15.0) | <0.001 | -18.4 (-20.0 to -16.8) | -18.9 (-20.6 to -17.3) | <0.001 |
| Global Radial strain (%) | 1004 | 36.6 (31.8-41.7) | 41.9 (36.8-47.3) | <0.001 | 42.7 (38.5-47.3) | 48.3 (43.4-53.5) | <0.001 |
| Global Circumferential strain (%) | 1004 | -18.3 (-20.5 to -16.1) | -20.4 (-22.8 to -18.3) | <0.001 | -21.6 (-23.3 to -19.9) | -23.7 (-25.6 to -21.9) | <0.001 |
| RVEDVi (ml/m$^2$) | 962 | 81 (72-91) | 65 (57-72) | <0.001 | 90 (80-100) | 76 (69-84) | <0.001 |
| RVESVi (ml/m$^2$) | 962 | 38 (33-46) | 29 (25-34) | <0.001 | 39 (34-46) | 31 (26-36) | <0.001 |
| RVEF (%) | 962 | 53 (48-57) | 55 (51-59) | <0.001 | 56 (52-60) | 59 (56-63) | <0.001 |
| LA volume index (ml/m$^2$) | 1458 | 36 (29-45) | 30 (24-36) | <0.001 | 40 (33-47) | 36 (30-43) | <0.001 |
| ***Blood tests*** | | | | | | | |
| Haemoglobin (g/L) | 1811 | 142 (134-151) | 142 (135-150) | 0.889 | 142 (133-151) | 141 (133-150) | 0.638 |
| Creatinine (mmol/L) | 2488 | 72 (62-82) | 68 (60-79) | <0.001 | 73 (63-82) | 70 (61-80) | <0.001 |
| Albumin (g/L) | 5606 | 45 (43-47) | 45 (43-46) | 0.357 | 45 (44-47) | 45 (44-47) | <0.001 |
| NT-proBNP (NPX) | 33 282 | -0.10 (-0.86-0.65) | -0.29 (-0.78-0.66) | 0.353 | -0.17 (-0.89-0.54) | -0.18 (-0.82-0.54) | 0.106 |
| HbA1c (mmol/mol) | 2705 | 35 (33-37) | 36 (34-38) | <0.001 | 34 (32-36) | 35 (33-37) | <0.001 |
| ***Comorbidities*** | | | | | | | |
| Hypertension (n[%]) | 0 | 632 (17.1) | 207 (26.7) | <0.001 | 1872 (12.0) | 4358 (24.1) | <0.001 |
| Ischaemic heart disease (n[%]) | 0 | 153 (4.1) | 20 (2.6) | 0.054 | 300 (1.9) | 327 (1.8) | 0.452 |
| Stroke/TIA (n[%]) | 0 | 36 (1.0) | 10 (1.3) | 0.545 | 118 (0.8) | 172 (1.0) | 0.063 |
| Diabetes mellitus (n[%]) | 0 | 105 (2.8) | 43 (5.6) | <0.001 | 219 (1.4) | 544 (3.0) | <0.001 |
| Atrial Fibrillation (n[%]) | 0 | 47 (1.3) | 10 (1.3) | 1.000 | 63 (0.4) | 53 (0.3) | 0.103 |
| Chronic liver disease (n[%]) | 0 | 1 (0.0) | 0 (0.0) | 1.000 | 23 (0.2) | 22 (0.1) | 0.617 |
| Chronic respiratory disease (n[%]) | 0 | 462 (12.5) | 122 (15.8) | 0.016 | 1808 (11.6) | 2018 (11.1) | 0.207 |
| Chronic kidney disease (n[%]) | 0 | 4 (0.1) | 1 (0.1) | 1.000 | 20 (0.1) | 26 (0.1) | 0.815 |
| ***Medications*** | | | | | | | |
| Aspirin (n[%]) | 0 | 476 (12.9) | 106 (13.7) | 0.568 | 1311 (8.4) | 1941 (10.7) | <0.001 |
| Beta-blockers (n[%]) | 0 | 176 (4.8) | 42 (5.4) | 0.485 | 454 (2.9) | 741 (4.1) | <0.001 |
| ACEi/ARB (n[%]) | 0 | 322 (8.7) | 79 (10.2) | 0.205 | 859 (5.5) | 1768 (9.8) | <0.001 |
| Calcium Channel Blockers (n[%]) | 0 | 147 (4.0) | 37 (4.8) | 0.351 | 488 (3.1) | 919 (5.1) | <0.001 |
| Loop Diuretic (n[%]) | 0 | 12 (0.3) | 4 (0.5) | 0.627 | 21 (0.1) | 49 (0.3) | 0.009 |
| Statin (n[%]) | 0 | 458 (12.4 | 127 (16.4) | 0.003 | 1169 (7.5) | 2094 (11.6) | <0.001 |

LVEF; left ventricular ejection fraction, CCI; cardiac contractility index, BMI; body mass index, Q; quartile, SBP; systolic blood pressure, LVEDVi; left ventricular end-diastolic volume index, LVESVi; left ventricular end-systolic volume index, RVEDVi; right ventricular end-diastolic volume index, RVESVi; right ventricular end-systolic volume index, RVEF; right ventricular ejection fraction, LA; left atrial, NT-proBNP; N-terminal B-type natriuretic peptide, HbA1c; glycosylated haemoglobin, TIA; transient ischaemic attach, ACEi; angiotensin converting enzyme inhibitor, ARB; angiotensin receptor blocker.

**Table S3**

Title: Age and sex adjusted Poisson regression model of the risk of incident heart failure in the UK Biobank, having excluded participants who had abnormal LVEF at baseline.

| Incident Heart Failure | |
|---|---|
| | **Adjusted HR (95% CI)** |
| Age (per year) | 1.13 (1.11-1.16) |
| Male | 1.78 (1.34-2.37) |
| CCI <4.4mmHg/ml/$m^2$ | 1.33 (1.01-1.75) |

HR; hazard ratio, CCI; cardiac contractility index.
